# What are the psychosocial problem areas, needs and resources in people with coexisting type 2 diabetes and schizophrenia? Results of a survey study

**DOI:** 10.1192/j.eurpsy.2025.669

**Published:** 2025-08-26

**Authors:** S. T. Rønne, M. J. Rothmann, R. Jørgensen, B. Cleal, R. I. Holt, P. H. Gæde, L. E. Joensen, S. M. Arnfred

**Affiliations:** 1 Research Unit of Psychiatry West, Region Zealand, Slagelse; 2 Department of Clinical Medicine, Faculty of Health and Medical Sciences, University of Copenhagen, Copenhagen; 3 Department of Clinical Research, University of Southern Denmark; 4 Steno Diabetes Center Odense, Odense University hospital, Odense; 5 Unit for Psychiatric Research, Aalborg University Hospital; 6 Department of Clinical Medicine, Aalborg University, Aalborg; 7 Steno Diabetes Center Copenhagen, Copenhagen University Hospital, Copenhagen, Denmark; 8 Human Development and Health, Faculty of Medicine, University of Southampton; 9 Southampton National Institute for Health Research Biomedical Research Centre, University Hospital Southampton NHS Foundation Trust, Southampton, United Kingdom; 10 Department of Cardiology and Endocrinology, Slagelse Hospital, Slagelse; 11 Department of Regional Health Research, University of Southern Denmark, Odense; 12 Psychiatric Research Unit, Region Zealand, Slagelse, Denmark

## Abstract

**Introduction:**

People with schizophrenia have a higher prevalence of type 2 diabetes than the background population due to adverse effects from antipsychotics, lifestyle, and genetics.

**Objectives:**

This study aims to explore the illness and treatment burden, mental well-being, and received support for illness management among people with schizophrenia and type 2 diabetes.

**Methods:**

62 Danish adults recruited from psychiatric outpatient clinics participated in this cross-sectional study using a survey developed for this specific purpose. The survey included measures of burden of illness and treatment (daily impact of diabetes and schizophrenia, multimorbidity treatment burden, diabetes empowerment), mental well-being (general well-being and diabetes distress), and social relations and support (general and illness-specific support). Descriptive analyses of survey data were conducted.

**Results:**

Participants reported very negative daily impact from living with schizophrenia. However, diabetes also negatively impacted physical health, emotional well-being, and feelings about their future. 55% reported high treatment burden, 74% reported low/moderate diabetes empowerment. Approximately 30% had high levels of diabetes distress and 49% reported low general well-being. Half of the participants reported needing support for managing type 2 diabetes equal to schizophrenia.

**Conclusions:**

Living with schizophrenia and type 2 diabetes often involves high burden of illness and treatment, low diabetes empowerment, high levels of diabetes distress and low general well-being. This study highlights a need for engaging mental health professionals, care coordinators, family and friends in daily diabetes management in future interventional studies and clinical practice.

**Disclosure of Interest:**

None Declared

